# circSLC6A6 Sponges miR-497-5p to Promote Endometrial Cancer Progression via the PI4KB/Hedgehog Axis

**DOI:** 10.1155/2021/5512391

**Published:** 2021-06-22

**Authors:** Juan Hu, Xing Peng, Weina Du, Yichuan Huang, Chun Zhang, Xian Zhang

**Affiliations:** ^1^Department of Obstetrics and Gynecology, The Central Hospital of Wuhan, Tongji Medical College, Huazhong University of Science and Technology, Wuhan, China; ^2^Intensive Care Unit, The Central Hospital of Wuhan, Tongji Medical College, Huazhong University of Science and Technology, Wuhan, China

## Abstract

**Background:**

As a new kind of noncoding RNAs, circular RNAs (circRNAs) have been substantiated to be involved in multiple biological processes. Accumulating studies indicate that circular RNAs (circRNAs) regulate the development of cancers by acting as miRNA sponges. However, the role of circRNAs in endometrial cancer (EC) is rarely reported. This study was aimed at investigating the functional roles of circSLC6A6 in EC.

**Methods:**

The qRT-PCR assay was performed to detect the circSLC6A6 expression in EC tissues and cell lines. The luciferase reporter assay was performed to explore the connection between circSLC6A6 and miR-497-5p as well as the connection between miR-497-5p and PI4KB. The colony formation assay, EdU assay, wound healing assay, and transwell assay were performed to examine the proliferation, migration, and invasion of EC cells. The in vivo assay was performed to reveal the function of circSLC6A6 in tumorigenesis.

**Results:**

We found that circSLC6A6 was highly expressed in both EC tissues and cells. And circSLC6A6 promoted the proliferation, migration, and invasion of EC cells in vitro. In vivo, circSLC6A6 promoted tumor growth. Besides, a mechanistic study demonstrated that circSLC6A6 could regulate tumor-associated signaling PI4KB/hedgehog pathway by sponging miR-497-5p.

**Conclusion:**

This study illustrates that circSLC6A6 plays a role in promoting EC progression via the miR-497-5p-mediated PI4KB/hedgehog pathway. Our study may provide a potential novel biomarker for EC diagnosis or treatment.

## 1. Introduction

Endometrial cancer (EC) is the fourth most common cancer in women in industrialized country, being classified into many subtypes containing endometrioid endometrial cancer, serous endometrial cancer, clear cell endometrial cancer, mixed endometrial cancer, and uterine carcinosarcoma [1, 2]. For patients diagnosed at early stages, the 5-year survival rate of them is up to 95% [3]. However, the 5-year survival rate of patients at advanced stages is less than 30% [4]. Although there are many therapies, such as surgery, chemotherapy, radiotherapy, and hormone therapy [5], the problems of chemotherapeutic resistance and poor response rate bring obstacles for the treatment [6, 7]. Therefore, it is urgent to explore the underlying molecular mechanism of EC pathogenesis.

Circular RNAs (circRNAs), a subclass of noncoding RNAs with high stability and sequence conservation, are widely expressed in cells and tissues [8, 9]. It has been reported that circRNAs are involved in the development of multiple tumors, such as gastric cancer, breast cancer, hepatocellular cancer, and endometrial cancer [10–14]. But the function of circRNAs in EC is still not clarified clearly.

Hedgehog (Hh) signaling, firstly discovered in *Drosophila* by genetic analysis, is highly conserved in evolution and function and essential in the development of normal embryos [15, 16]. And the abnormal activation of Hh signaling promotes the cancer progression [17–19]. Phosphatidylinositol 4-kinase III*β* (PIK4B), regulating cellular physiological functions that contain cell morphology, metabolic regulation, and signal transduction, can produce PI4P that participates in Hh signaling in the dictyosome [17]. And the disruption of signaling transduction from PTC to SMO that controls PI4P is considered the most common feature in the tumor [20]. Consequently, the PIK4B/hedgehog pathway might be involved in the pathogenesis of EC.

In our study, we explored the function and regulatory mechanism of circSLC6A6 in EC. Firstly, it was reported that circSLC6A6 was upregulated in EC and promoted EC cell proliferation, migration, and invasion. In addition, we found that the function of circSLC6A6 in EC was associated with the miR-497-5p-mediated PIK4B/hedgehog pathway. These findings indicate that circSLC6A6 might serve as a potential biomarker for EC.

## 2. Materials and Methods

### 2.1. Tissue Samples and Cell Lines

The frozen EC tissues and normal tissues were obtained from the Central Hospital of Wuhan. All patients presented their written consent.

293T cells, hESC cells, and EC cells (ISK, HEC-1A, HEC-1B, and KLE) were obtained from the China Center for Type Culture Collection (CCTCC, Wuhan, China). ISK, HEC-1B, and KLE cells were cultured in Dulbecco's Modified Eagle's Medium (DMEM; Gibco, USA). The HEC-1A cell was cultured in McCoy's 5A Medium. All media were supplemented with 10% fetal bovine serum (FBS; Gibco, USA), 100 U/mL penicillin, and 100 *μ*g/mL streptomycin. Cells were cultured under 5% CO_2_ at 37°C.

### 2.2. RNA Extraction and qRT-PCR

Total RNA was extracted from EC tissues and cell lines by using the Trizol reagent (Invitrogen, USA). The concentration of RNA was measured applying NanoDrop 2000 (Thermo, USA). Reverse-transcription PCR was performed using PrimeScript RT Master Mix (Takara, Japan). The qRT-PCR was performed using SYBR Premix Ex Taq and SYBR PrimeScript miRNA RT-PCR Kit (Takara, Japan). For circRNA and mRNA, GAPDH was used as an internal control. For miRNA, U6 was applied as an internal control. The reactions were run on the ABI 7500 Real-Time PCR System (Life Technologies, USA). The relative expression levels of RNAs were calculated with the 2^−ΔΔCT^ algorithm. The primers used in this study are as follows:

*circSLC6A6-F*: 5′-CAGCTAGCGGTGTATGCCTT-3′

*circSLC6A6-R*: 5′-ATGCCCCCTTCAGAGGTGTA-3′

*miR-497-5p (stem-loop RT primer)*: 5′-GTCGTATCCAGTGCAGGGTCCGAGGT

ATTCGCACTGGATACGACACCAAACA-3′

*miR-497-5p-F*: 5′-TGCGCCAGCAGCACACTGTGG-3′

*miR-497-5p-R*: 5′-CAGTGCGTGTCGTGGAGT-3′

*PI4KB-F*: 5′-ATGCCACTGCCAGCATAAGT-3′

*PI4KB-R*: 5′-ATACTCTCGGTGCTGGAGGA-3′

*Hedgehog-F*: 5′-GACCGCGACCGCAATAAGTA-3′

*Hedgehog-R*: 5′-GCGGCTCACCGGACTTGA-3′

*GAPDH-F*: 5′-GAGAAGTATGACAACAGCCTC-3′

*GAPDH-R*: 5′-ATGGACTGTGGTCATGAGTC-3′

*U6-F*: 5′-CTCGCTTCGGCAGCACATATACTA-3′

*U6-R*: 5′-ACGAATTTGCGTGTCATCCTTGCG-3′

### 2.3. RNase R Treatment

10 *μ*g total RNA from HEC-1A cells was incubated with 5 U/*μ*g RNase R (YEASEN, China) for 10 minutes at 37°C. Then, the RNase R-treated RNA was reversely transcribed to cDNA and quantified by the qRT-PCR assay.

### 2.4. Cell Transfection

si-circSLC6A6-1, si-circSLC6A6-2, miR-497-5p mimics, and miR-497-5p inhibitors and their controls were purchased from RiboBio (Guangzhou, China); oe-circSLC6A6 plasmids were constructed using pLO-ciR vectors. They were transfected into HEC-1A and HEC-1B cells using Lipofectamine 3000 (Invitrogen, USA). The sequences are listed in Table S1.

### 2.5. Luciferase Reporter Assay

Luciferase reporter plasmids (pGL3) containing circSLC6A6-MT, circSLC6A6-MuT, PI4KB-WT, or PI4KB-WuT sequences were cotransfected with miR-497-5p mimics or NC mimics into 293T cells. After 24 h, cells were lysed by 1× PLB; then, the luciferase activities were examined by the Dual-Luciferase Reporter Assay System (Promega, USA).

### 2.6. RNA Immunoprecipitation (RIP) Assay

The RIP assay was performed following using Magna RIP™ RNA-binding Protein Immunoprecipitation Kit (Millipore, USA) as in previous reports [21]. RIP Lysis Buffer was used to lyse the HEC-1A and HEC-1B cells; then, the lysis solution was incubated with the magnetic beads conjugated with anti-Argonaute 2 (AGO2) or anti-IgG antibodies for 6 h. The RNA pull-down was quantified by the qRT-PCR assay.

### 2.7. EdU Assay

The EdU assay was performed using the Cell-Light EdU Apollo567 In Vitro Kit (RiboBio, China). 2 × 10^5^ HEC-1A and HEC-1B cells were seeded into 96-well plates and cultured for 24 h. Then, the cells were incubated with 20 *μ*M EdU solution for 2 h and were fixed using 4% paraformaldehyde. Hoechst was used to stain cells. The pictures were captured by using an Olympus microscope.

### 2.8. Colony Formation Assay

500 HEC-1A or HEC-1B cells were seeded into 6-well plates and cultured for 2 weeks. Then, the cells were fixed using 4% paraformaldehyde and stained using crystal violet for 15 min. The colony numbers in plates were counted.

### 2.9. Wound Healing Assay

HEC-1A cells or HEC-1B cells were seeded into 6-well plates and cultured for 24 h. The 200 *μ*L pipette tips were used to scratch the cells. Then, the EC cells were cultured with medium containing 2% FBS for 48 h. The pictures were captured at 0 h and 48 h. And the relative migration rate was presented by the diminishing distance.

### 2.10. Transwell Assay

Transwell chambers (Corning, USA) were used to examine the invasion or migration of EC cells. The medium containing EC cells without FBS was seeded into the upper chamber. The medium containing 10% FBS was infused into the bottom chamber as a cell chemoattractant. After 24 h of incubation, the cells on the upper chamber were fixed with 4% paraformaldehyde and then stained with crystal violet for 10 min. The cells on the upper chamber were counted.

### 2.11. Western Blot

EC cells were lysed by RIPA buffer containing protease inhibitor. Total protein extracted from EC cells was separated by sodium dodecyl sulphate-polyacrylamide gel electrophoresis and transferred to polyvinylidene fluoride (PVDF) membranes (Millipore, USA). The membranes were blocked with 5% skimmed milk and incubated with the primary antibodies (1 : 1000, Abcam, USA) overnight at 4°C. The secondary antibodies (1 : 10000, Abcam, USA) were incubated for 1 h at room temperature. Then, the Clarity Western ECL Substrate (Bio-Rad, USA) was used to visualize the protein bands.

### 2.12. Xenografts in Mice

All 12 male BALB/c nude mice (four-week-old, 18-20 g) were obtained from the Wuhan Center for Disease Control and Prevention. 1 × 10^7^ HEC-1B cells stably transfected with si-circSLC6A6 (*n* = 6) or controls (*n* = 6) were subcutaneously injected into the mice, respectively. Tumor volumes were measured every 7 days. After 28 days, the tumors were excised, measured, and photographed. Then, PI4KB and hedgehog expressions in tumors were measured with immunohistochemical (IHC) staining using anti-PI4KB and anti-hedgehog antibodies.

### 2.13. Statistical Analysis

GraphPad Prism 8.0 (GraphPad Software, USA) and SPSS 23.0 (IBM, Chicago, USA) were used in this study. Student's *t*-test and one-way ANOVA were used to analyze the significance between groups. *p* < 0.05 was considered statistically significant.

## 3. Results

### 3.1. circSLC6A6 Is Upregulated in Endometrial Cancer

**Figure 1:**
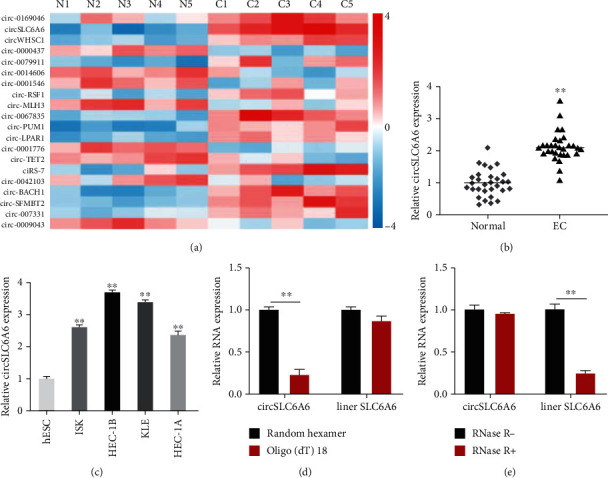
circSLC6A6 is upregulated in endometrial cancer: (a) the heat map of dysregulated circRNAs in 5 endometrial cancer tissues using circRNA sequencing; (b) the expression of circSLC6A6 in 30 pairs of EC tissues and matched normal tissues by qRT-PCR; (c) the expression of circSLC6A6 in EC cell lines by qRT-PCR; (d) the expressions of circSLC6A6 and linear SLC6A6 amplified by random hexamer and oligo(dt)18; (e) the expressions of circSLC6A6 and linear SLC6A6 after RNase R treatment (^∗∗^*p* < 0.01).

We screened the expression profiles of circRNAs taken from 5 EC and adjacent noncancerous endometrial tissues using circRNA sequencing. The significantly dysregulated circRNAs were presented in the heat map (Figure 1(a)). It was found that circ_0064428 (termed circSLC6A6 in the remainder of the article) was the most upregulated circRNA. To verify whether circSLC6A6 expression was upregulated in EC tissues, we detected the expression of circSLC6A6 in 30 pairs of EC tissues using qRT-PCR; the results in Figure 1(b) showed that circSLC6A6 levels were significantly increased in EC tissues. Moreover, circSLC6A6 expressions in EC cell lines were significantly higher than those in the normal cell line (Figure 1(c)). HEC-1A and HEC-1B were the two cell lines with the lowest and largest change of circSLC6A6 expression separately and then were selected for subsequent studies. To verify the circular feature of circSLC6A6, random hexamer and oligo(dt)18 were used to amplify circSLC6A6 and linear SLC6A6, respectively (Figure 1(d)). The RNase R treatment assay indicated that circSLC6A6 was resistant to RNase R, while linear LDLR mRNA was distinctly digested by RNase R, which confirmed the circular features of circSLC6A6 (Figure 1(e)). These results showed that circSLC6A6 is an upregulated circular RNA in EC.

### 3.2. circSLC6A6 Promotes EC Cell Proliferation, Migration, and Invasion In Vitro

**Figure 2:**
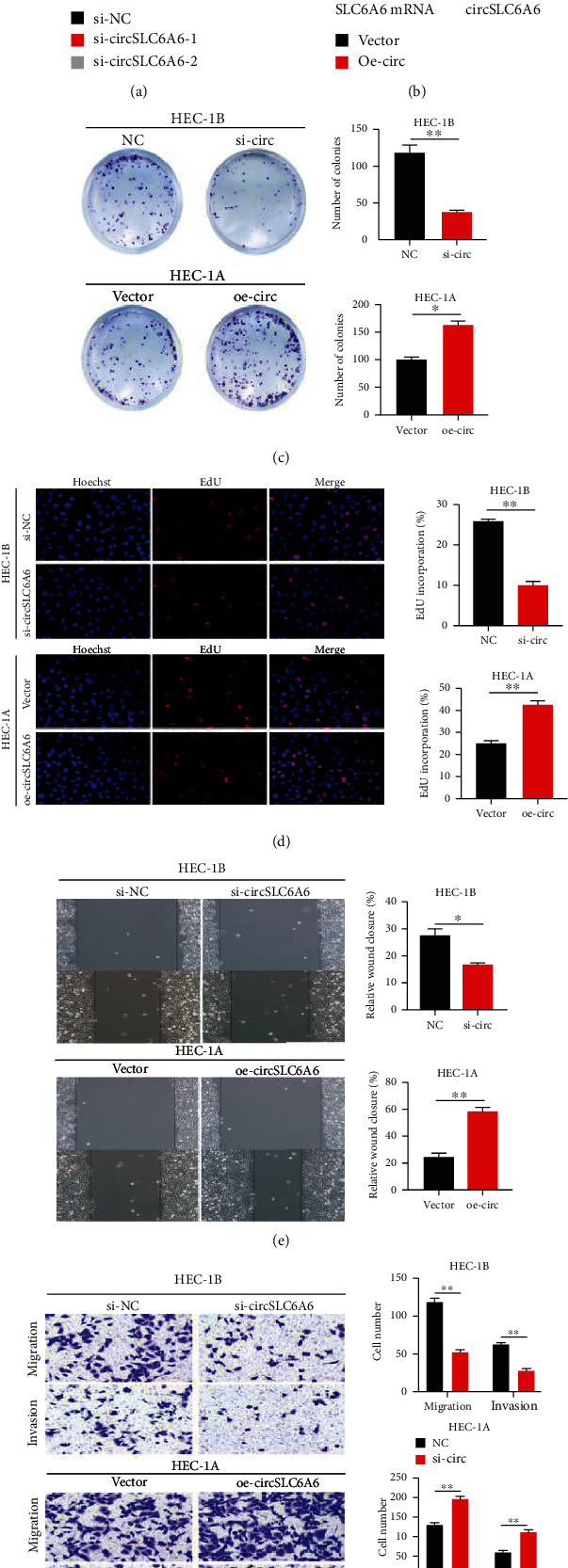
circSLC6A6 promotes EC cell proliferation, migration, and invasion in vitro. (a) The relative circSLC6A6 expression in HEC-1B cells transfected with si-circSLC6A6-1 and si-circSLC6A6-2, respectively, by qRT-PCR. (b) The relative circSLC6A6 expression in HEC-1A cells transfected with oe-circSLC6A6 vectors by qRT-PCR. (c) The proliferation abilities of HEC-1B cells transfected with si-circSLC6A6 and HEC-1A cells transfected with oe-circSLC6A6 were determined by the colony formation assay. (d) The proliferation abilities of HEC-1B cells transfected with si-circSLC6A6 and HEC-1A cells transfected with oe-circSLC6A6 were determined by the EdU assay. (e) Cell migration was detected by the wound healing assay in HEC-1B cells transfected with si-circSLC6A6 and HEC-1A cells transfected with oe-circSLC6A6. (f) Cell migration and invasion abilities of HEC-1B cells transfected with si-circSLC6A6 and HEC-1A cells transfected with oe-circSLC6A6 were detected by the transwell assay (^∗^*p* < 0.05, ^∗∗^*p* < 0.01).

To explore the function of circSLC6A6 in EC cells, two siRNAs against circSLC6A6 and the overexpression vector of circSLC6A6 were constructed. It showed that si-circSLC6A6-1 has higher inhibitory efficiency in the HEC-1B cell and was selected for the subsequent experiments in Figure 2(a). The overexpression vector of circSLC6A6 in HEC-1A cells significantly induced the expression of circSLC6A6 (Figure 2(b)). The colony formation assay showed that circSLC6A6 knockdown significantly decreased the colonies of HEC-1B cell, whereas circSLC6A6 upregulation in the HEC-1A cell exerted opposite effects (Figure 2(c)). The EdU assay demonstrated that the EdU-positive cells were obviously reduced by the circSLC6A6 downregulation and greatly increased by circSLC6A6 overexpression (Figure 2(d)). In addition, the wound healing assay (Figure 2(e)) and transwell assay (Figure 2(f)) revealed that circSLC6A6 downregulation suppressed the migration and invasion of EC cells, whereas the impairment of migration and invasion was blocked by circSLC6A6 upregulation. These data indicate that circSLC6A6 promotes the proliferation, migration, and invasion of EC cells.

### 3.3. circSLC6A6 Serves as an Efficient miR-497-5p Sponge in EC Cells

**Figure 3:**
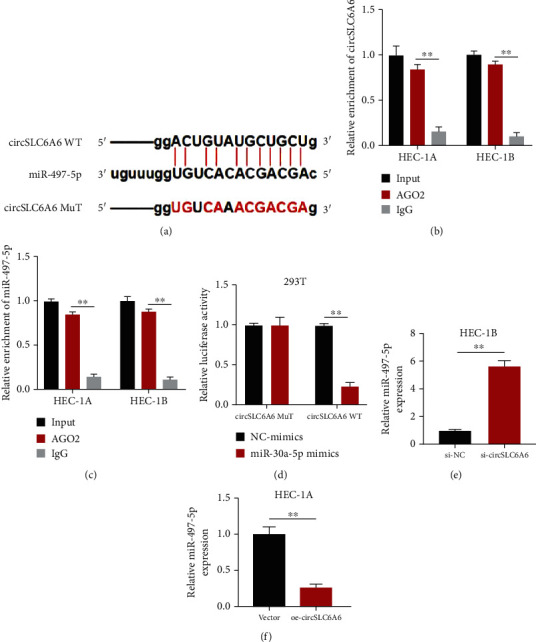
circSLC6A6 serves as an efficient miR-497-5p sponge in EC. (a) The putative binding sites between circSLC6A6 WT/MuT and miR-497-5p. (b, c) RIP experiments with the AGO2 antibody were conducted to detect the expression of circSLC6A6 and miR-497-5p in HEC-1A and HEC-1B cells. (d) Luciferase activity was detected in luciferase reporter vectors harboring circSLC6A6 WT/MuT sequences and miR-497-5p mimic-/NC mimic-cotransfected 293T cells. (e, f) The expression of miR-497-5p in HEC-1B cells transfected with si-circSLC6A6 and HEC-1A cells transfected with oe-circSLC6A6 was detected using the qRT-PCR assay (^∗∗^*p* < 0.01).

It was reported that circRNAs can abrogate the function of miRNAs via acting as miRNA sponges [22]. To further explore the downstream pathway of circSLC6A6 in EC, it was predicted that miR-497-5p was the potential target miRNA of circSLC6A6 by CircInteractome (Figure 3(a)). The RIP assay was performed to pull down the RNA transcripts that are bound to AGO2 in HEC-1B cells transfected with miR-497-5p mimics or NC mimics. The results showed that circSLC6A6 and miR-497-5p were pulled down by anti-AGO2 antibodies, suggesting that circSLC6A6 interacted with miR-497-5p through AGO2 protein in EC cells (Figures 3(b) and 3(c)). Next, the luciferase report assay showed that luciferase activity was significantly reduced in circSLC6A6 wild-type and miR-497-5p mimic groups, indicating that circSLC6A6 can directly interact with miR-497-5p in Figure 3(d). In addition, the expressions of miR-497-5p were significantly upregulated in EC cells transfected with si-circSLC6A6 but downregulated in EC cells transfected with oe-circSLC6A6 (Figures 3(e) and 3(f)). These results demonstrate that circSLC6A6 can serve as a miR-497-5p sponge in EC cells.

### 3.4. miR-497-5p Mediates the PI4KB/Hedgehog Pathway in EC Cells

**Figure 4:**
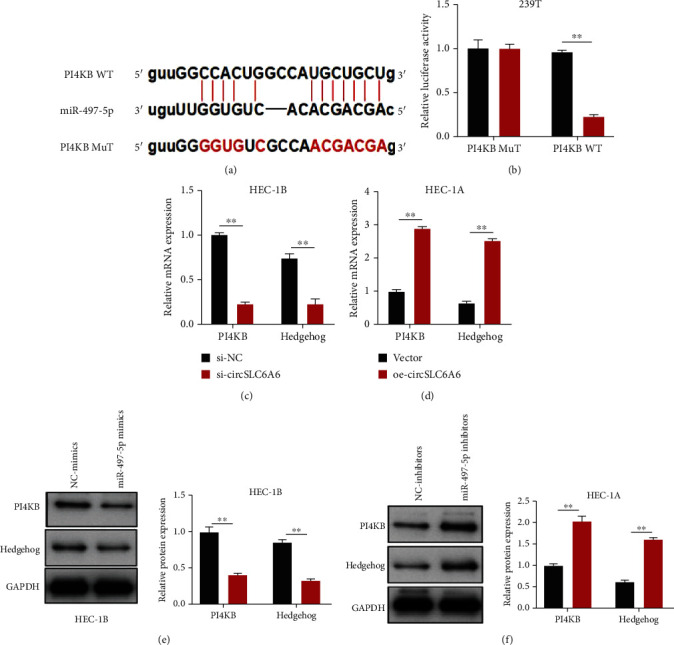
miR-497-5p mediates the PI4KB/hedgehog pathway in EC. (a) The putative binding sites between PI4KB WT/MuT and miR-497-5p. (b) Luciferase report assay was performed to verify the interaction between PI4KB and miR-497-5p in 293T cells (black column—NC mimics, red column—miR-497-5p mimics). (c, d) The expressions of PI4KB and hedgehog in HEC-1B cells transfected with si-circSLC6A6 and HEC-1A cells transfected with oe-circSLC6A6 were detected by qRT-PCR. (e) The expressions of PI4KB and hedgehog in HEC-1B cells transfected with miR-497-5p mimics were detected by western blotting (black column—NC mimics, red column—miR-497-5p mimics). (f) The expressions of PI4KB and hedgehog in HEC-1A cells transfected with miR-497-5p inhibitors were detected by western blotting (black column—NC mimics, red column—miR-497-5p inhibitors) (^∗∗^*p* < 0.01).

The potential target genes of miR-497-5p were predicted using TargetScan and Starbase. Among them, PI4KB was reported to be involved in the progression of cancers [23]. In this study, PI4KB was selected as the target gene of miR-497-5p for further exploration. Hedgehog signaling is upregulated in basal cell carcinoma and medulloblastoma, and PI4KB may be a relevant target protein for pharmacological inhibition of Hh signaling [17]. The binding sites of miR-497-5p and PI4KB are shown in Figure 4(a). The luciferase report assay in Figure 4(b) verified that miR-497-5p mimics reduced luciferase activity in the PI4KB wild-type group, whereas no suppression was observed in the PI4KB mutant group. In addition, the qRT-PCR assay showed that circSLC6A6 knockdown would significantly reduce the expressions of PI4KB and hedgehog in HEC-1B cells, but circSLC6A6 overexpression would significantly increase the expressions of PI4KB and hedgehog in HEC-1A cells in Figures 4(c) and 4(d). Western blotting in Figures 4(e) and 4(f) showed that miR-497-5p mimics remarkably decreased the expression of PI4KB and hedgehog in HEC-1B cells, while miR-497-5p inhibitors enhanced the expression of PI4KB and hedgehog in HEC-1A cells. These data reveal that miR-497-5p mediates the PI4KB/hedgehog pathway in EC cells.

### 3.5. circSLC6A6 Promotes EC Cell Proliferation, Migration, and Invasion via miR-497-5p/PIK4B/Hedgehog

**Figure 5:**
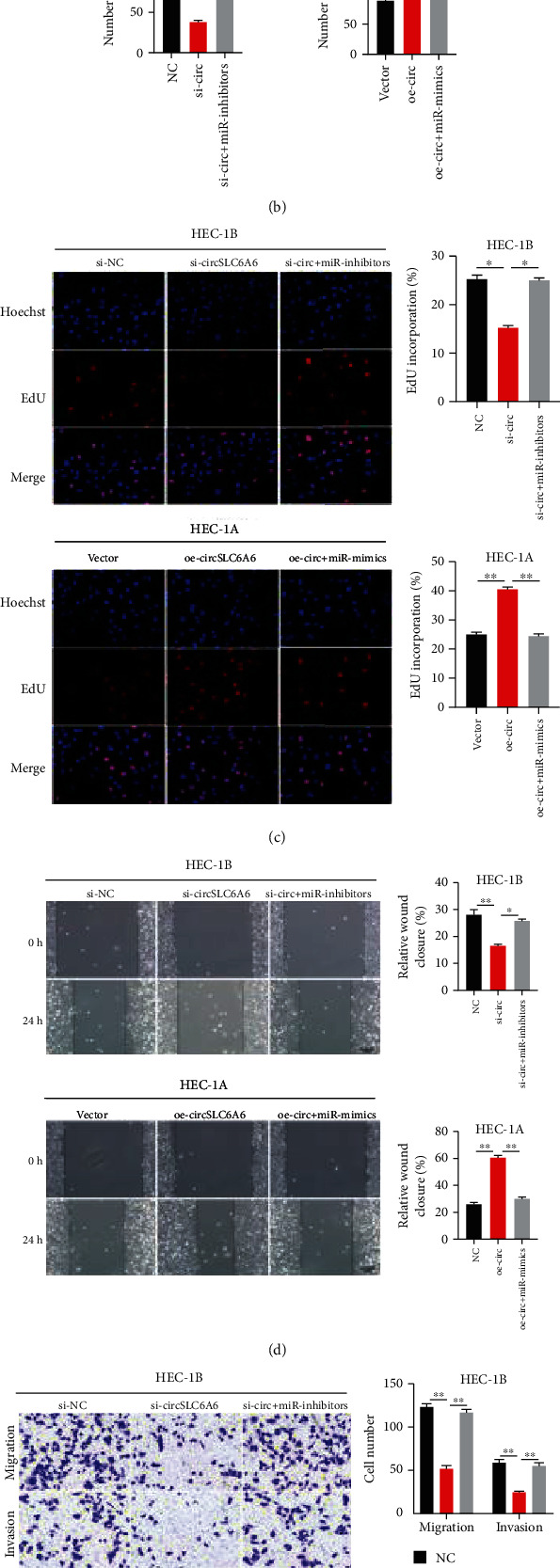
circSLC6A6 promotes EC cell proliferation, migration, and invasion via miR-497-5p/PIK4B/hedgehog. (a) The expressions of PI4KB and hedgehog in HEC-1B and HEC-1A cells were detected by western blot. (b, c) The proliferation abilities of HEC-1B and HEC-1A cells by the colony formation assay and EdU assay. (d) Wound healing assay was conducted to detect the migration of treated EC cells. (e) The migration and invasion abilities of HEC-1B and HEC-1A cells were detected using the transwell assay. (f) The expressions of GLI1, PTCH, and SHH proteins in HEC-1B and HEC-1A cells (^∗∗^*p* < 0.01).

Then, the results in Figure 5(a) revealed that the downregulation of circSLC6A6 significantly increased miR-497-5p expression and decreased the expressions of PIK4B/hedgehog, and the upregulation of circSLC6A6 markedly suppressed miR-497-5p expression and enhanced PIK4B/hedgehog expression, whereas miR-497-5p inhibitors or mimics reversed the above effects, respectively. The colony formation assay (Figure 5(b)) and EdU assay (Figure 5(c)) showed that circSLC6A6 inhibition reduced HEC-1B cell proliferation and circSLC6A6 overexpression promoted HEC-1A cell proliferation, but extra supplement of miR-497-5p inhibitors or mimics could block the effects induced by circSLC6A6 inhibition or overexpression. The wound healing assay (Figure 5(d)) and transwell assay (Figure 5(e)) indicated that circSLC6A6 advanced the migration and invasion of EC cells, while miR-497-5p could attenuate the circSLC6A6-induced effects. And western blotting showed that miR-497-5p inhibition in EC cells would promote the expressions of downstream targets GLI1, PTCH, and SHH in the hedgehog pathway, but extra supplement of si-PI4KB in EC cells transfected with the miR-497-5p inhibitor would in turn decrease the expressions of GLI1, PTCH, and SHH (Figure 5(f)). These data suggest that circSLC6A6 promotes EC cell proliferation, migration, and invasion via miR-497-5p/PIK4B/hedgehog.

### 3.6. Silencing circSLC6A6 Inhibits the Growth of Tumors In Vivo

**Figure 6:**
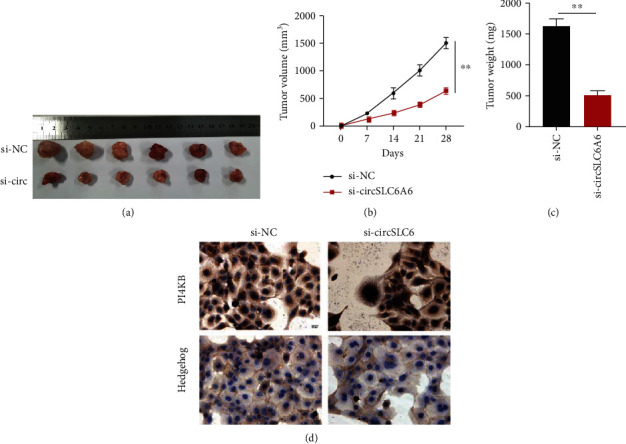
Silencing circSLC6A6 inhibits the growth of tumors in vivo. (a) The image of xenograft tumors was shown. (b) Tumor volumes were recorded every 7 days. (c) Tumor weights were recorded. (d) The PI4KB and hedgehog expressions were explored in EC tumor tissues by IHC (^∗∗^*p* < 0.01).

To further investigate the effects of circSLC6A6 on tumor growth in vivo, HEC-1B cells stably transfected with si-circSLC6A6 or si-NC were subcutaneously injected into BALB/c nude mice. The image of xenograft tumors is shown in Figure 6(a). It was shown that tumor volumes were significantly decreased via circSLC6A6 inhibition with the growth of days (Figure 6(b)). Tumor weights of the si-circSLC6A6 group are significantly reduced in Figure 6(c). The IHC assay showed that the PIK4B and hedgehog expressions were downregulated in the tumor of the si-circSLC6A6 group, as shown in Figure 6(d). The results reveal that the inhibition of circSLC6A6 suppressed the tumor growth of EC in vivo.

## 4. Discussion

Increasing studies have shown that circRNAs act as regulators of multiple biological processes, especially the cancer progression [24]. However, studies focusing on circRNAs in EC are little to understand. In the present study, we analyzed the circRNA expression profiles using circRNA sequencing and found that circSLC6A6 was the most upregulated circRNA in EC. Then, it was found that the expression levels of circSLC6A6 in EC tissues and cell lines were significantly increased, which was consistent with the data analysis. In addition, functional experiments showed that circSLC6A6 promoted the cell proliferation, migration, and invasion in vitro as well as tumor growth in vivo.

Currently, cancer-related studies are focusing on the regulatory role of “circRNA-miRNA-mRNA” axis; many researches have reported that circRNAs can regulate gene expression by sequestering miRNAs that mediate the metastasis, metabolism, and proliferation of tumor cells [24, 25]. Therefore, the potential miRNA target miR-497-5p of circSLC6A6 was predicted by CircInteractome. The results of the RIP assay and luciferase report assay indicated that circSLC6A6 sponged miR-497-5p in EC cells. In addition, PI4KB was predicted to be the target gene of miR-497-5p by Starbase. PI4KB is reported to be involved in the activation of Hh signaling, which is closely associated with cancer development [15]. Subsequently, the mechanistic study confirmed that miR-497-5p mediated the PI4KB/Hh pathway in EC cells. In this study, we first revealed the oncogenic effects of the PI4KB/Hh pathway in EC. We demonstrated that circSLC6A6 promoted EC cell proliferation, migration, and invasion by regulating the PI4KB/Hh pathway through miR-497-5p.

However, there are several limitations in this study. The premise of noncoding RNA as an essential biomarker is that it can be stably detected in body fluids such as plasma [21]; therefore, whether circSLC6A6 can be detected in body fluids needs further investigation. In addition, the connection between circSLC6A6 and EC stages also needs further study and we will continue to investigate these issues.

In conclusion, the present research provides the evidence that circSLC6A6 contributes to EC development. circSLC6A6/miR-497-5p/PI4KB/Hh signaling might be a potential direction for EC diagnosis and therapy.

## Data Availability

The data of this study are available from the corresponding author on reasonable request.

## Supplementary Materials

Supplementary Materials Supplementary Table S1: the sequences of si-RNAs and miR-mimics/inhibitors.

## References

[1] Di Tucci C., Capone C., Galati G. (2019). Immunotherapy in endometrial cancer: new scenarios on the horizon. *Journal of Gynecologic Oncology*.

[2] Urick M. E., Bell D. W. (2019). Clinical actionability of molecular targets in endometrial cancer. *Nature Reviews. Cancer*.

[3] Siegel R. L., Miller K. D., Jemal A. (2019). Cancer statistics, 2019. *CA: a Cancer Journal for Clinicians*.

[4] Su L., Wang H., Miao J., Liang Y. (2015). Clinicopathological significance and potential drug target of CDKN2A/p16 in endometrial carcinoma. *Scientific Reports*.

[5] Hüsing A., Dossus L., Ferrari P. (2016). An epidemiological model for prediction of endometrial cancer risk in Europe. *European Journal of Epidemiology*.

[6] Davidson B. A., Foote J., Clark L. H. (2016). Tumor grade and chemotherapy response in endometrioid endometrial cancer. *Gynecol Oncol Rep.*.

[7] Kong J., He X., Wang Y., Li J. (2019). Effect of microRNA-29b on proliferation, migration, and invasion of endometrial cancer cells. *The Journal of International Medical Research*.

[8] Jeck W. R., Sharpless N. E. (2014). Detecting and characterizing circular RNAs. *Nature Biotechnology*.

[9] Chen L. L. (2016). The biogenesis and emerging roles of circular RNAs. *Nature Reviews. Molecular Cell Biology*.

[10] Liu W., Zhao J., Jin M., Zhou M. (2019). circRAPGEF5 contributes to papillary thyroid proliferation and metastatis by regulation miR-198/FGFR1. *Mol Ther Nucleic Acids.*.

[11] Xu H., Gong Z., Shen Y., Fang Y., Zhong S. (2018). Circular RNA expression in extracellular vesicles isolated from serum of patients with endometrial cancer. *Epigenomics*.

[12] Liu Y., Chen S., Zong Z. H., Guan X., Zhao Y. (2020). CircRNA WHSC1 targets the miR-646/NPM1 pathway to promote the development of endometrial cancer. *Journal of Cellular and Molecular Medicine*.

[13] Zong Z. H., Liu Y., Chen S., Zhao Y. (2020). Circ_PUM1 promotes the development of endometrial cancer by targeting the miR-136/NOTCH3 pathway. *Journal of Cellular and Molecular Medicine*.

[14] Liu Y., Chang Y., Cai Y. (2020). Hsa_circ_0061140 promotes endometrial carcinoma progression via regulating miR-149-5p/STAT3. *Gene*.

[15] Skoda A. M., Simovic D., Karin V., Kardum V., Vranic S., Serman L. (2018). The role of the Hedgehog signaling pathway in cancer: a comprehensive review. *Bosnian Journal of Basic Medical Sciences*.

[16] Ma Y., Erkner A., Gong R. (2002). Hedgehog-mediated patterning of the mammalian embryo requires transporter-like function of dispatched. *Cell*.

[17] Kremer L., Hennes E., Brause A. (2019). Discovery of the Hedgehog pathway inhibitor Pipinib that targets PI4KIIIß. *Angewandte Chemie (International Ed. in English)*.

[18] Yavari A., Nagaraj R., Owusu-Ansah E. (2010). Role of lipid metabolism in smoothened derepression in hedgehog signaling. *Developmental Cell*.

[19] Bhateja P., Cherian M., Majumder S., Ramaswamy B. (2019). The hedgehog signaling pathway: a viable target in breast cancer?. *Cancers (Basel).*.

[20] Rohatgi R., Scott M. P. (2007). Patching the gaps in Hedgehog signalling. *Nature Cell Biology*.

[21] Wei H., Yan S., Hui Y. (2020). CircFAT1 promotes hepatocellular carcinoma progression via miR-30a-5p/REEP3 pathway. *Journal of Cellular and Molecular Medicine*.

[22] Wu Y., Xie Z., Chen J. (2019). Circular RNA circTADA2A promotes osteosarcoma progression and metastasis by sponging miR-203a-3p and regulating CREB3 expression. *Molecular Cancer*.

[23] Morrow A. A., Alipour M. A., Bridges D., Yao Z., Saltiel A. R., Lee J. M. (2014). The lipid kinase PI4KIII*β* is highly expressed in breast tumors and activates Akt in cooperation with Rab11a. *Molecular Cancer Research*.

[24] Zhang Z., Yang T., Xiao J. (2018). Circular RNAs: promising biomarkers for human diseases. *eBioMedicine*.

[25] Hu Q., Du K., Mao X., Ning S. (2018). miR-197 is downregulated in cervical carcinogenesis and suppresses cell proliferation and invasion through targeting forkhead box M1. *Oncology Letters*.

