# Supplementary material for: circSLC6A6 Sponges miR-497-5p to Promote Endometrial Cancer Progression via the PI4KB/Hedgehog Axis
Source: J Immunol Res. 2021 Jun 22;2021:5512391. doi: 10.1155/2021/5512391 (PMC8245255; doi:10.1155/2021/5512391)
Supplement: Supplementary Materials — Supplementary Table S1: the sequences of si-RNAs and miR-mimics/inhibitors. [file 5512391.f1.docx]

Supplementary Material

Table S1. The sequences of si-RNAs, miR-mimics/inhibitors.

| Name | Sequences |
| --- | --- |
| si-circSLC6A6-1 | 5’-GGATATATGGTGCGTTTCTCA-3’ |
| si-circSLC6A6-2 | 5’-ATATATGGTGCGTTTCTCATA-3’ |
| si-NC | 5’-CAGTCACGTTAATGGTCGTT-3’ |
| miR-497-5p mimics | 5’-CAGCAGCACACUGUGGUUUGUAAACCACAGUGU  GCUGCUGUU-3’ |
| miR-497-5p inhibitors | 5’-ACAAACCACAGUGUGCUGCUG-3’ |
| NC-mimics | 5’-UUCUCCGAACGUGUCACGUTT-3’ |
| NC-inhibitors | 5’-CAGUACUUUUGUGUAGUACAA-3’ |
